# Synthesis and Characterization of Inorganic-Organic Derivatives of Layered Perovskite-like Niobate HSr_2_Nb_3_O_10_ with *n*-Amines and *n*-Alcohols

**DOI:** 10.3390/molecules28124807

**Published:** 2023-06-16

**Authors:** Alina D. Khramova, Oleg I. Silyukov, Sergei A. Kurnosenko, Ekaterina N. Malygina, Irina A. Zvereva

**Affiliations:** Department of Chemical Thermodynamics and Kinetics, Institute of Chemistry, Saint Petersburg State University, 198504 Saint Petersburg, Russia; st062003@gmail.com (A.D.K.); st040572@student.spbu.ru (S.A.K.); st805605@student.sbpu.ru (E.N.M.); irina.zvereva@spbu.ru (I.A.Z.)

**Keywords:** layered compounds, perovskite, niobate, inorganic-organic hybrid, intercalation, grafting

## Abstract

A protonated and hydrated Dion-Jacobson-phase HSr_2_Nb_3_O_10_∙yH_2_O was used to prepare two series of inorganic–organic derivatives containing non-covalently intercalated *n*-alkylamines and covalently grafted *n*-alkoxy groups of different lengths, as they are promising hybrid materials for photocatalytic applications. Preparation of the derivatives was carried out both under the conditions of standard laboratory synthesis and by solvothermal methods. For all the hybrid compounds synthesized structure, quantitative composition, a type of bonding between inorganic and organic parts as well as light absorption range were discussed using powder XRD, Raman, IR and NMR spectroscopy, TG, elemental CHN analysis, and DRS. It was shown that the inorganic–organic samples obtained contain approximately one interlayer organic molecule or group per proton of the initial niobate, as well as some amount of intercalated water. In addition, the thermal stability of the hybrid compounds strongly depends on the nature of the organic component anchoring to the niobate matrix. Although non-covalent amine derivatives are stable only at low temperatures, covalent alkoxy ones can withstand heat up to 250 °C without perceptible decomposition. The fundamental absorption edge of both the initial niobate and the products of its organic modification lies in the near-ultraviolet region (370–385 nm).

## 1. Introduction

Perovskite-like oxides with a layered structure have been intensively studied both experimentally and theoretically due to the unique physicochemical properties exhibited by individual representatives of this class of compounds, such as high catalytic and photocatalytic activity [1], high-temperature superconductivity [2], and colossal magnetoresistance [3]. Most layered perovskite-like oxides capable of ion-exchange reactions [4] can be transformed into their protonated forms [5,6], which, on the one hand, are proton conductors [7,8] and photocatalysts for water splitting [1,9] and, on the other hand, exhibit the ability to intercalate water [10,11] and other molecules [12,13], give derivatives with grafted organic modifiers [14,15,16,17], and undergo exfoliation into nanolayers [18,19]. Materials based on layered oxides are promising objects for use as high-temperature ion conductors in fuel cells [20], catalysts in industrial reactions [21], as well as in devices for water and air purification [22,23], microelectronics [24], and photovoltaics [25].

Several niobium-based perovskite-like layered oxides with the general formula AB_2_Nb_3_O_10_ (where A = alkali metal and B = alkaline earth metal), related to the Dion–Jacobson phases, have been intensively studied since the synthesis of compounds of the composition ACa_2_Nb_3_O_10_ (A = Li, Na, K) [26]. The crystal structure of KSr_2_Nb_3_O_10_ is similar to that of the KCa_2_Nb_3_O_10_ phase. Liang was the first to describe the synthesis of KSr_2_Nb_3_O_10_ by the solid-phase method [27]. This layered perovskite-like oxide is capable of ion exchange [27,28], intercalation [29,30], and its derivatives exhibit superconductivity properties [31].

The protonated form of the oxide (HSr_2_Nb_3_O_10_∙yH_2_O) can be obtained from the alkaline form KSr_2_Nb_3_O_10_ by ion exchange in acids [27]. In the protonated compound HSr_2_Nb_3_O_10_, the layers are stacked to give an eight-coordinated interlayer site, while in the initial alkaline form, they are displaced by half a unit cell to give a six-coordinated interlayer site. This is because adjacent layers in the potassium compound are stacked to optimize the coordination of the interlayer cations [32].

It was reported that protonated compounds HSr_2_Nb_3_O_10_∙yH_2_O are capable of intercalating organic bases, such as alkylamines [33] and pyridine [32], grafting some *n*-alcohols (MeOH, PrOH, HxOH) [34] and also reacting readily with alkyldiamines (C_2_-C_12_) [29]. In addition, the protonated form can be exfoliated into nanolayers with a perovskite structure via the introduction of bulk organic bases (TBAOH), as a rule, followed by a physical action (shaking or sonication) [35,36,37].

Currently, the reactivity of the protonated niobate HSr_2_Nb_3_O_10_∙yH_2_O with organic substances has not been sufficiently studied, except for some of the papers presented above. However, the formation of its organically modified derivatives deserves special attention since the inorganic–organic hybrids based on layered perovskite-like oxides are of high interest as high-performance photocatalytic materials for hydrogen production and water purification, outperforming the initial compounds in the activity up to several orders of magnitude [38,39,40,41,42,43,44].

**Figure 1:**
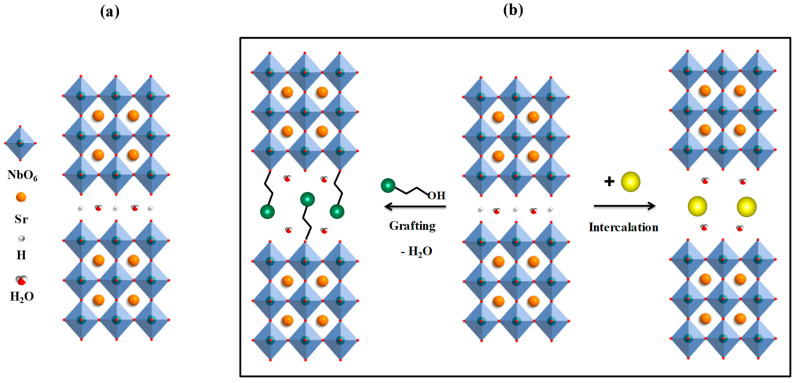
The schematic view of structure HSr_2_Nb_3_O_10_∙yH_2_O (**a**) and intercalation and grafting processes (**b**).

With that said, this work is devoted to the synthesis and systematic study of two series of the simplest inorganic–organic derivatives of the niobate HSr_2_Nb_3_O_10_∙yH_2_O (Figure 1a) containing intercalated (inserted between perovskite blocks of the layered structure) *n*-alkylamines and grafted (covalently bound to perovskite blocks) *n*-alkoxy groups of alcohols with various chain lengths (Figure 1b). Special attention is paid to the structural features of hybrid compounds, their quantitative composition, thermal stability, and light absorption.

## 2. Results and Discussion

### 2.1. Preparation of Inorganic–Organic Derivatives

Here and further in the text, formulas of the form HSN_3_ × RNH_2_ and HSN_3_ × ROH will be used for the inorganic–organic derivatives of HSr_2_Nb_3_O_10_∙yH_2_O (HSN_3_∙yH_2_O) with *n*-alkylamines and *n*-alcohols (R = methyl Me, ethyl Et, *n*-propyl Pr, *n*-butyl Bu, *n*-hexyl Hx, *n*-octyl Oc, and *n*-decyl Dc).

To determine the possibility of obtaining the derivatives with *n*-amines and *n*-alcohols and optimize the synthesis conditions, several series of experiments were carried out with variable temperature (25–200 °C), duration (1–14 d), and concentration of the organic component (38–100%). The possibility of obtaining long-chain derivatives was investigated using previously obtained derivatives as precursors with shorter organic modifiers, such as BuNH_2_, OcNH_2_, MeOH, and PrOH. In addition, some syntheses were carried out under hydrothermal-microwave conditions, which were also varied to reduce the duration of the experiment.

Estimated optimized conditions for obtaining inorganic–organic derivatives are given below in Table 1.

### 2.2. Identification of the Initial Alkaline and Protonated Niobate

The results of powder X-ray phase analysis (XRD) were used to identify the initial alkaline KSr_2_Nb_3_O_10_ (KSN_3_) and protonated HSr_2_Nb_3_O_10_·yH_2_O (HSN_3_·yH_2_O) forms of the niobate and calculate their structural parameters (Figure S1). According to the results of the X-ray phase analysis, the alkaline and protonated forms are single-phase compounds, not containing a noticeable amount of impurities, and their structural parameters (tetragonal *a* = 3.89 Å, *c* = 29.6 Å for the alkaline and *a* = 3.89 Å, *c* = 16.4 Å for the protonated one) are in good agreement with the literature data [27]. Due to the process of water intercalation during the formation of HSN_3_·yH_2_O from KSN_3_, the interlayer distance *d* (the distance between the centers of neighboring perovskite plates) increases. It was noted that the protonated hydrated form HSN_3_·yH_2_O quickly becomes dehydrated in air, and, in connection with this, it is rather difficult to fix the exact water content per formula unit. According to the TG analysis, the water content in the resulting HSN_3_·yH_2_O varies from 0.5 to 1.6 water molecules per structural unit, and the degree of potassium substitution with protons was found to be close to 100%.

### 2.3. Characterization of Inorganic–Organic Derivatives

**Figure 2:**
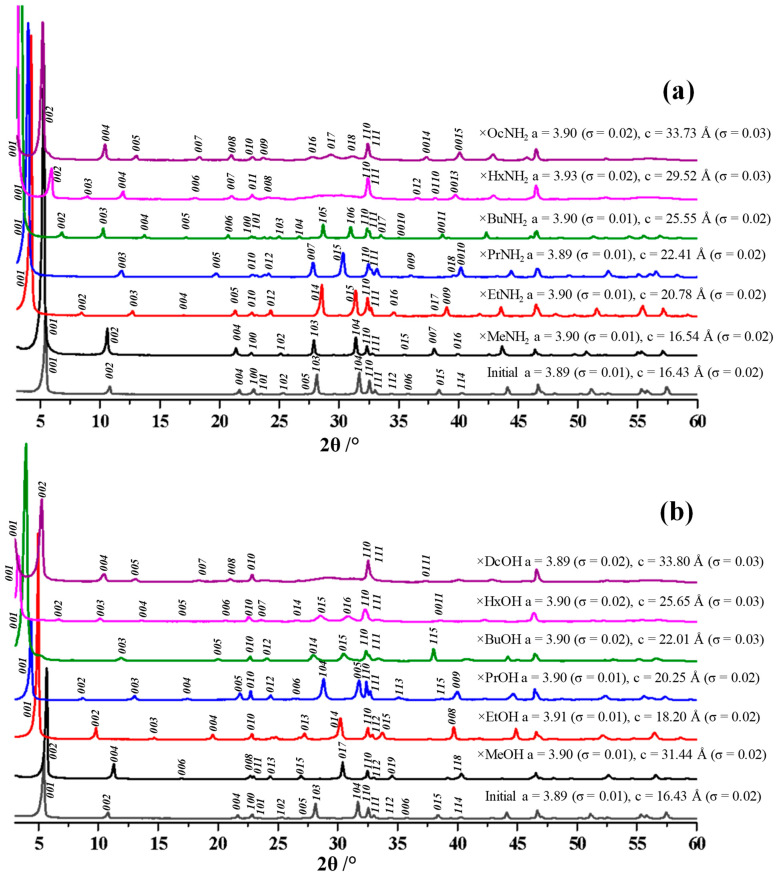
XRD patterns of the initial niobate HSN_3_·yH_2_O and its inorganic–organic derivatives with *n*-amines HSN_3_ × RNH_2_ (**a**) and *n*-alcohols HSN_3_ × ROH (**b**) with tetragonal unit cell parameters and their standard deviations σ.

Results of the X-ray phase analysis (Figure 2) indicate the successful preparation of target single-phase products of the organic modification. All the reflections observed in the diffraction patterns of the hybrid compounds can be indexed in the tetragonal system. The intercalation of amines and the grafting of alcohols into the interlayer space leads to its significant expansion, which is manifested by a characteristic shift of the *(00x)* reflections to the low-angle region. This shift is proportional to the length of the organic chain. In this case, the lattice parameter *a* practically does not change, which indicates that the sizes of the niobium-oxygen octahedra remain unchanged. X-ray diffraction patterns of all the inorganic–organic compounds (except methanol derivative) are indexed without doubling the *c* parameter, which indicates the likely preservation of the eclipsed configuration of the adjacent perovskite slabs.

**Figure 3:**
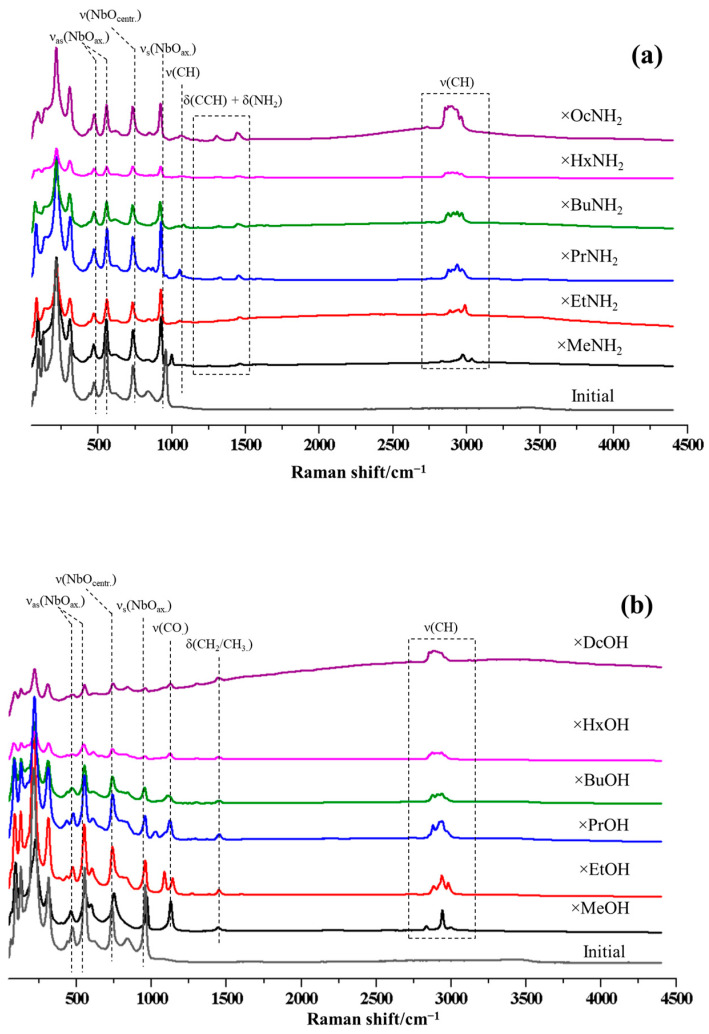
Raman spectra of the initial niobate HSN_3_·yH_2_O and its inorganic–organic derivatives with *n*-amines HSN_3_ × RNH_2_ (**a**) and *n*-alcohols HSN_3_ × ROH (**b**).

Successful formation of the amine and alcohol derivatives is also demonstrated by the appearance in their Raman spectra (Figure 3) of characteristic bands corresponding to the vibrational frequencies of the organic component and absent in the case of the initial protonated form HSN_3_·yH_2_O. In particular, deformation vibrations of C–C–H (1330–1340 cm^–1^), CH_2_/CH_3_ (1450–1460 cm^–1^), NH_2_ (1570–1580 cm^–1^), as well as stretching of C–N (1030–1070 cm^–1^), C–O (1040–1070 cm^–1^), and C–H (2800–3050 cm^–1^) bonds are observed. However, what is most important in the case of alkoxy derivatives is the absence of vibrations of OH-groups, which may indicate the covalent bonding of *n*-alkoxy fragments and not the intercalation of molecular alcohols. In addition, the introduction of organic structures into the interlayer space causes a shift of the band of symmetric vibrations of the Nb–O axial double bond to the low-frequency region in the case of intercalated amines while in the case of alcohols, there are two bands in the spectrum, one corresponding to the Nb–O axial double bond almost without changes and another corresponding to the Nb–O–R significantly shifted to the low-frequency region (around 750 cm^−1^). These shifts are manifestations of interatomic interactions in the space between the layers. The bands corresponding to vibrations of the central octahedrons in three-layer niobates (771 cm^−1^) change insignificantly.

In addition, the analysis of the bands of stretching and bending vibrations of *n*-alkylamine and *n*-alkoxy fragments observed in the IR spectra of the samples (Figure S2) also confirmed the successful intercalation and grafting of the organic molecules.

**Figure 4:**
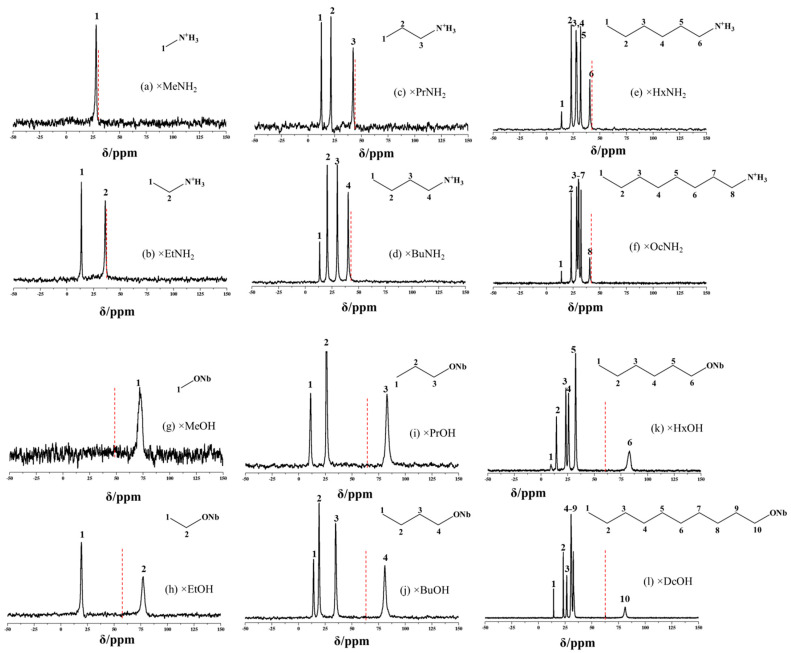
^13^C NMR spectra of inorganic–organic derivatives with *n*-amines HSN_3_ × RNH_2_ (**a**–**f**) and *n*-alcohols HSN_3_ × ROH (**g**–**l**). The position of the signal from the carbon (1) for the pure liquid amines and alcohols is indicated by a red dotted line.

Solid-state ^13^C NMR spectra of the samples shown in Figure 4 not only confirm the successful preparation of the hybrids but also allow the establishment of the nature of bonding between the niobate matrix and organic modifiers. The intercalation of amines is accompanied by a slight shift (~2 ppm) of the bands, belonging to the carbon atoms nearest to the amino group, in the direction of a high field in comparison with the spectra of molecular amines, which indicates that the introduced amine molecules exist in the cationic form. At the same time, the fact of the formation of a covalent bond in alkoxy derivatives is confirmed by a shift of the bands belonging to the carbon atom nearest to the oxygen by approximately 16–19 ppm into the region of a low field in comparison with their position in the spectra of molecular alcohols. In the case of long-chain alkoxy derivatives (HSN_3_ × BuOH, HSN_3_ × HxOH, HSN_3_ × DcOH) prepared on the basis of the *n*-propanol derivative (HSN_3_ × PrOH), ^13^C NMR confirms the absence of residual *n*-propoxy groups in the samples. The spectra of the long-chain derivatives in all cases show some band overlapping due to similar chemical shifts.

**Figure 5:**
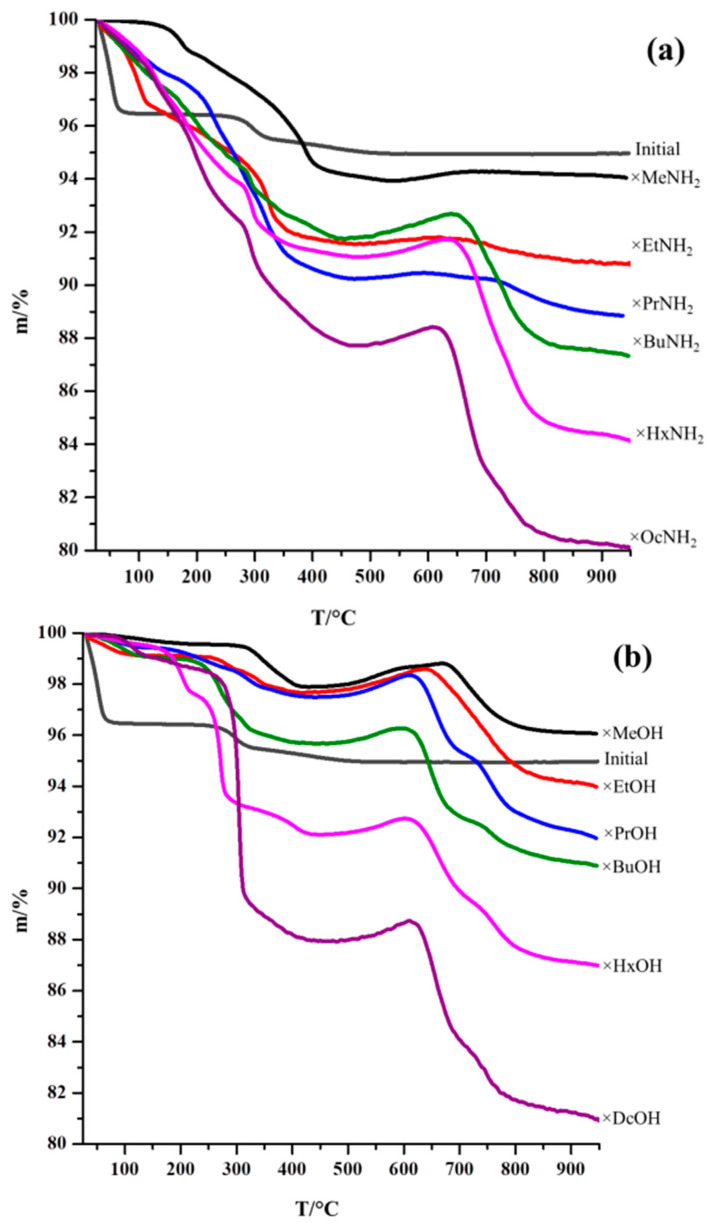
TG curves of the initial niobate HSN_3_·yH_2_O and inorganic–organic derivatives with *n*-amines HSN_3_ × RNH_2_ (**a**) and *n*-alcohols HSN_3_ × ROH (**b**).

TG curves of the original protonated niobate and its inorganic–organic derivatives measured in an oxidizing atmosphere are shown in Figure 5.

In the case of the protonated form, two main segments can be distinguished on the curve. The first of them, corresponding to temperatures up to 100 °C, refers to the deintercalation of interlayer water molecules with the formation of the anhydrous protonated niobate. In the second section (about 250–600 °C), topochemical dehydration occurs, i.e., the release of protons bound with interlayer oxygen anions. Thus, the final products of thermolysis have an overall formula Sr_2_Nb_3_O_9,5_. The thermal decomposition of organically modified niobates is more complex. It includes the stage of the release of intercalated water and partially amine molecules at relatively low temperatures, the decomposition stage of the hybrid as such (usually near the decomposition temperature of the original protonated form), and the stage of oxidation of residual carbon-containing compounds (accompanied by the weight gain) followed by the complete combustion of residual carbon [45]. In the case of the inorganic–organic derivatives with methylamine and methanol, increased stability is observed, and the start of decomposition is shifted to higher temperatures, relative to the derivatives with longer chains. Similar behavior was previously observed for other layered oxides [39,40].

**Figure 6:**
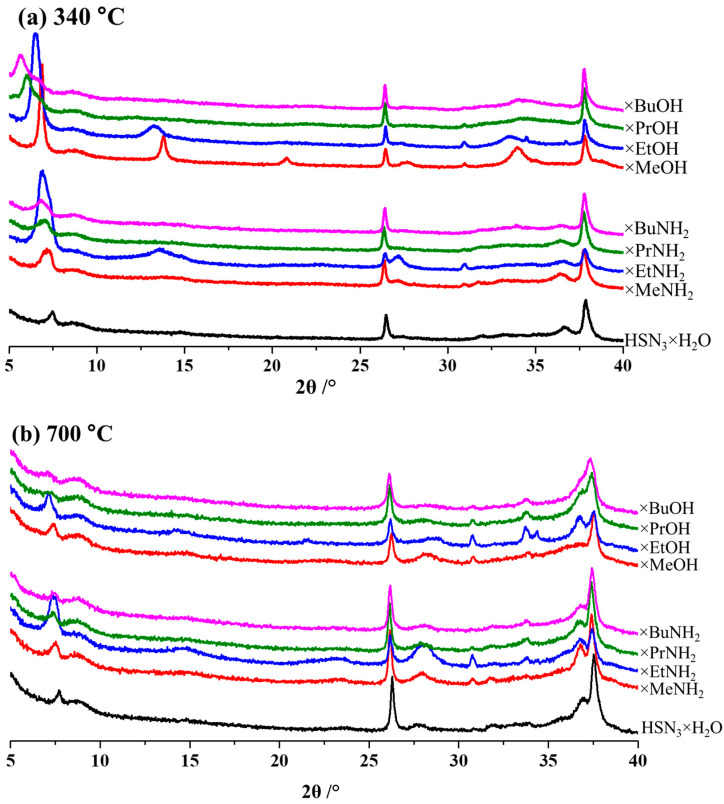
Data of thermal X-ray phase analysis of the initial protonated niobate HSN_3_∙yH_2_O and its derivatives of *n*-amines HSN_3_ × RNH_2_ and *n*-alcohols HSN_3_ × ROH at 340 °C (**a**) and 700 °C (**b**).

Analyzing the thermal X-ray diffraction analysis (Figure S4b–e) obtained for the initial protonated form and several derivatives with organics, it can be concluded that the decomposition of amine derivatives proceeds as amine deintercalation from the interlayer space, as a result of which, at temperatures above 200 °C, a dehydrated form of layered oxide begins to appear (in this case, the methylamine sample is noticeably resistant to the deintercalation process), the dehydration of which subsequently begins at a temperature of about 300 °C, and leads to the formation of the same products with an interlayer distance slightly higher than for the dehydrated protonated form at the same temperature (Figure 6a). Subsequent heating to 700 °C (Figure 6b) does not lead to a change in the structure of the samples. Thus, it can be concluded that the area of mass increase and decrease observed on the TG curves of amine derivatives (Figure 6a), most likely, refers to the oxidation of soot formed on the sample surface during the amine elimination.

In the case of the alcohol derivatives (Figure S4f–i), however, the increased stability was observed and XRD patterns do not significantly change upon heating up to 280 °C (except for the *n*-propanol derivative, for which heating to 100 °C causes noticeable structural changes, most likely associated with the release of interlayer water. This was also observed for the ethylamine derivative). A further increase in temperature leads to the formation (based on the analysis of the position of intense reflections in the small-angle region) of phases containing residual alkyl organic chains, which remain in the interlayer space up to the beginning of the oxidation region above 450–500 °C (Figure 5a). The diffraction patterns of the samples heated up to 700 °C (Figure 5b) look approximately the same and correspond to the pyrolysis products of the initial protonated form. This indicates that, as in the case of amine derivatives, there is practically no organic matter left in the interlayer space by that time, which is consistent with the TG data (Figure 6b).

In general, the decomposition process can be represented by the following reaction:

HSr_2_Nb_3_O_10_∙yH_2_O∙xOM → Sr_2_Nb_3_O_9,5_ + (y + 0.5)H_2_O↑ + xOM↑

where OM is the organic part (with general composition C_n_H_2n+1_NH_2_ for amines and C_n_H_2n_ for grafted alcohols) which either leaves the sample as a result of evaporation or as a result of oxidation to gaseous products. Using the TG data, the total weight loss of the hybrids upon heating in the air was calculated. Since the final compound after pyrolysis is Sr_2_Nb_3_O_9_._5_ and the mass of the organic part in the sample is known from CHN analysis, it is possible to calculate the amount of water released as a result of the decomposition of the protonated form (y) and the number of intercalated organics (x). Quantitative compositions of the hybrids determined by elemental CHN analysis and TG in this way are presented in Table 2.

**Figure 7:**
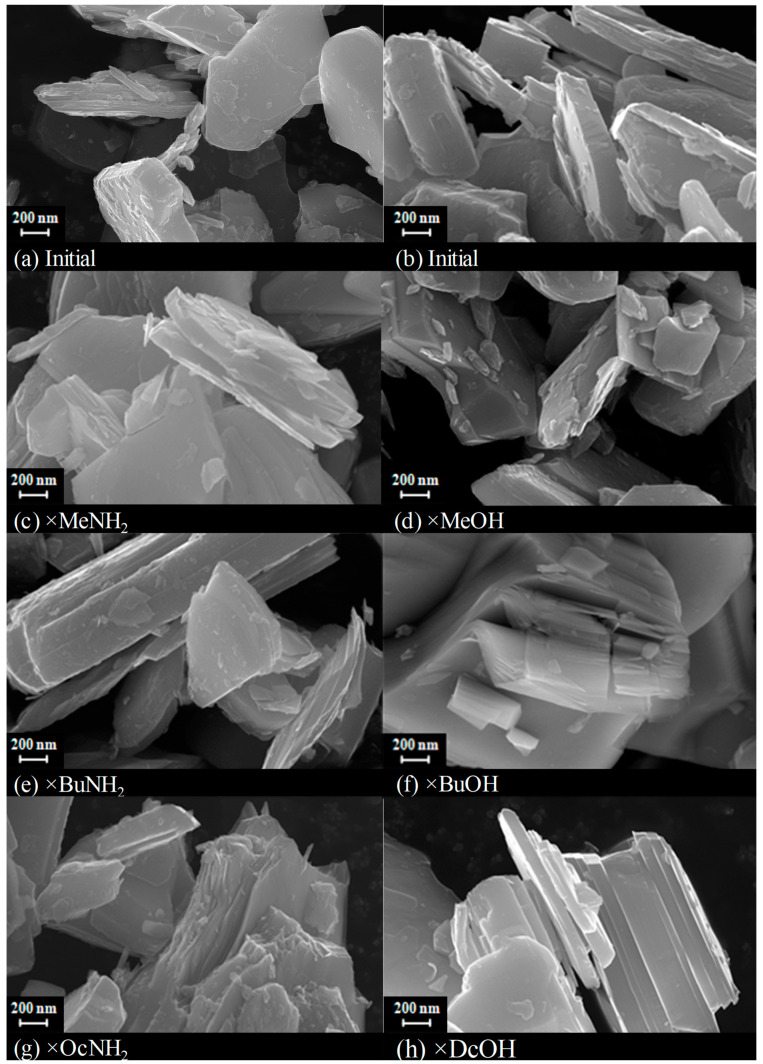
SEM images of the initial protonated niobate HSN_3_∙yH_2_O (**a**,**b**) and its derivatives with *n*-amines HSN_3_ × RNH_2_ (**c**,**e**,**g**) and *n*-alcohols HSN_3_ × ROH (**d**,**f**,**h**).

As can be seen from the SEM images (Figure 7), the introduction of organic molecules did not lead to significant changes in the morphology of lamellar particles of the layered oxide. However, for long-chain compounds, such as the decanol derivative, partial delamination of the oxide can be noted.

Since the inorganic–organic derivatives of HSN_3_ are of interest as promising photocatalytic materials, the corresponding samples were also studied using DRS to determine the optical range of their operation (Figure S3). The fundamental absorption edge of both the initial niobate and products of its organic modification lies in the near-ultraviolet region (370–385 nm) and approaches very close to the visible spectrum boundary in the case of short-chain derivatives with *n*-alcohols (Table 2). When moving from the initial niobate to the products of *n*-amine intercalation, the bandgap energy slightly grows with the increase in the interlayer distance *d* from 3.29 eV (HSN_3_∙yH_2_O) to 3.32 eV (HSN_3_ × BuNH_2_) and stays practically unchanged upon the further elongation of the *n*-amine chain length. During methanol grafting, the bandgap width, on the contrary, reduces to 3.22 eV, which appears to be caused by the influence of the covalently bonded organic modifier on the energy structure of the layered niobate matrix. However, the subsequent elongation of the *n*-alcohol length leads to a gradual increase in the bandgap width up to 3.34 eV (HSN_3_ × DcOH). This trend is quite expected since the bandgap energy of layered perovskite-like oxides usually rises with the interlayer space expansion [46]. In general, the HSN_3_-based inorganic–organic samples demonstrate a 0.2–0.3 eV lower bandgap width in comparison with the related derivatives of the HCa_2_Nb_3_O_10_ niobate [39,40] and, therefore, may use a greater part of solar irradiation to drive photocatalytic reactions.

## 3. Materials and Methods

### 3.1. Solid-Phase Synthesis of the Alkaline Form KSr_2_Nb_3_O_10_ (KSN_3_)

Synthesis of the layered perovskite-like niobate KSr_2_Nb_3_O_10_ (KSN_3_) was carried out by the ceramic method in the air. The SrCO_3_, Nb_2_O_5_, and K_2_CO_3_ were used as starting reagents. All the substances were pre-calcined to remove traces of moisture. The amounts of the reagents were taken in accordance with the stoichiometry of the solid-phase reaction but K_2_CO_3_ was taken in a 30% excess, since it partially sublimates and melts out of the pellet at high synthesis temperatures:

K_2_CO_3_+ 3Nb_2_O_5_ + 4SrCO_3_ → 2KSr_2_Nb_3_O_10_ + 5CO_2_

Weights of the initial substances, taken with an accuracy of 1 × 10^−4^ g, were thoroughly mixed and ground for 9 repetitions of 10 min each, with 5 min breaks on a planetary ball mill (Idar-Oberstein, Germany) in *n*-heptane at a rotation speed of 600 rpm. The mixture obtained was pelletized under a pressure of 50 atm in tablets of 0.5 g, which were placed in corundum crucibles and calcined in a muffle furnace (Lilienthal, Germany) at a temperature of 1300 °C for 10 h.

### 3.2. Preparation of the Protonated and Hydrated Form of HSr_2_Nb_6_O_10_ (HSN_3_·yH_2_O)

The protonated hydrated form of the niobate HSr_2_Nb_3_O_10_·yH_2_O (HSN_3_·yH_2_O) was obtained by ion exchange in an acid solution in accordance with the equation:

KSr_2_Nb_3_O_10_ + HNO_3_ + yH_2_O → HSr_2_Nb_3_O_10_·yH_2_O + KNO_3_

To do this, the tablets of KSN_3_ were carefully ground in an agate mortar, the resulting powder was transferred into a flask and filled with an excess of 6 M nitric acid. The suspension was stirred at room temperature for 48 h, then the solid phase was separated by centrifugation (Elmi CM-6MT centrifuge), washed with water, and dried over CaO for 24 h.

### 3.3. Synthesis of Inorganic–Organic Derivatives

Intercalation of *n*-amines was carried out by a direct one-stage reaction in closed glass vessels with stirring or under hydrothermal conditions in laboratory autoclaves. All amine derivatives were obtained directly from the protonated hydrated niobate. To do this, 250 mg of HSN_3_·yH_2_O was placed in a glass tube or a sealed 50 mL PTFE vessel of a steel laboratory autoclave with 25 mL of the corresponding amine solution and heated according to the appropriate temperature program (Table 1).

The synthesis of short-chain *n*-alkoxy derivatives HSN_3_ × ROH (R = Me, Et, Pr) was carried out based on the protonated form HSN_3_·yH_2_O, while the long-chain ones (R = Bu, Hx, Dc) were prepared from HSN_3_ × PrOH under hydrothermal conditions. In each case, 250 mg of the chosen precursor was placed in a sealed 50 mL PTFE vessel of a steel laboratory autoclave containing 25 mL of the alcohol or its solution and heated according to the appropriate temperature program (Table 1).

The resulting target products were filtered and washed with appropriate volatile solvents to remove residual amines and alcohols adsorbed on the surface. For this, acetone and *n*-hexane were chosen, which can provide the appropriate solubility of short-chain or long-chain organic components, respectively.

### 3.4. Instrumentation

#### 3.4.1. XRD

Phase composition and structure of the samples were studied using a Rigaku Miniflex II desktop diffractometer (Tokyo, Japan) using CuK_α_ radiation. X-ray diffraction (XRD) patterns were taken in the range of 2θ = 3–60° at a scanning rate of 10°/min. The resulting patterns were indexed using literature data and the powder diffraction database of The International Center for Diffraction Data (ICDD). The tetragonal lattice parameters were determined using the DiffracPlus Topas 4.2 software.

#### 3.4.2. Thermo-XRD

Thermo-XRD analysis was carried out using a research complex based on a Rigaku “Ultima IV” diffractometer with a Rigaku “R-300” low-temperature attachment and a Rigaku “SHT-1500” high-temperature attachment (Tokyo, Japan). The samples were taken in the temperature range from 28 °C to 1100 °C at a rate of 10 °C/min in an air atmosphere. The temperature step was 20 °C.

#### 3.4.3. Raman Spectroscopy

Raman spectra were recorded using a Bruker Senterra spectrometer (Billerica, MA, USA). The spectra were collected in the frequency range of 50−4000 cm^−1^, using a 488 nm laser (power 0.4–20 mW, accumulation time 10–250 s) as a radiation source.

#### 3.4.4. IR Spectroscopy

Infrared (IR) spectra were recorded using an IR Fourier-transform spectrometer Shimadzu IRAffinity-1 (Tokyo, Japan). The spectra were collected in the frequency range of 400−4000 cm^−1^ after preliminary tableting each sample in KBr.

#### 3.4.5. NMR Spectroscopy

Carbon nuclear magnetic resonance (^13^C NMR) spectra of the organically modified samples were recorded on a Bruker Avance III 400 WB spectrometer (Billerica, MA, USA) using tetramethylsilane as a standard.

#### 3.4.6. TG Analysis

Thermogravimetric (TG) analysis was performed on a Netzsch TG 209 F1 Libra thermobalance (Selb, Germany). Analysis of both the protonated niobate and its inorganic–organic derivatives was carried out in an oxidizing atmosphere (synthetic air) for the complete oxidation of organic substances located in the interlayer space. Each sample was heated from room temperature to 950 °C at a rate of 10 °C/min, followed by isothermal holding for 20 min.

#### 3.4.7. CHN Analysis

Carbon, hydrogen, and nitrogen content in the organically modified samples was determined via the elemental CHN analysis on a Euro EA3028-HT analyzer (Pavia, Italy).

#### 3.4.8. SEM

The morphology of the particles was investigated on a Zeiss Merlin scanning electron microscope (SEM) (Oberkochen, Germany) equipped with a field emission cathode, electron optics column Gemini II and an oil-free vacuum system.

#### 3.4.9. DRS

Diffuse reflectance spectra (DRS) were obtained on a Shimadzu UV-2550 spectrophotometer (Kyoto, Japan) equipped with an ISR-2200 integrating sphere in the range of 220–800 nm using barium sulfate as an external reference with reflection coefficient R = 1. The reflectance spectra were transformed into coordinates (F·hν)^1/2^ = f (hν), where F = (1 − R)^2^/2R is the Kubelka–Munk function. Linear sections of the graph were extrapolated and an optical bandgap energy E_g_ was found as an abscissa of their intersection point.

## 4. Conclusions

The protonated and hydrated Dion–Jacobson-phase HSr_2_Nb_3_O_10_∙yH_2_O can form inorganic–organic hybrids with *n*-alkylamines (*n* = 1–4, 6, 8) and *n*-alcohols (*n* = 1–4, 6, 10) containing non-covalently intercalated *n*-alkylamines and covalently grafted *n*-alkoxy groups of different lengths as interlayer modifiers. All the amine derivatives considered in the work can be obtained by direct reaction between the initial protonated oxide and corresponding alkylamine. In the case of derivatives with alcohols, direct grafting without by-products turned out to be possible only for the first three members of the series (*n* = 1–3), while the rest of the derivatives could be obtained using the propanol derivative as the initial one. The inorganic–organic samples obtained contain approximately one interlayer organic molecule or group per proton of the initial niobate as well as some amount of intercalated water. The thermal stability of the hybrid compounds strongly depends on the nature of the organic component. Although non-covalent amine derivatives are stable only at low temperatures < 80–100 °C (while methylamine derivative demonstrates quite high stability up to 180 °C), covalent alkoxy ones can withstand heat up to 250 °C without perceptible decomposition.

The fundamental absorption edge of both the initial niobate and products of its organic modification lies in the near-ultraviolet region (370–385 nm) and approaches very close to the visible spectrum boundary in the case of short-chain derivatives with *n*-alcohols. The inorganic–organic derivatives of HSr_2_Nb_3_O_10_ obtained exhibit 0.2–0.3 eV lower bandgap energies as compared to those of the related HCa_2_Nb_3_O_10_-based samples studied earlier, which allows them to utilize a greater part of solar irradiation in the photocatalytic reactions.

On the whole, it can be said that the resulting hybrids based on the layered Dion–Jacobson strontium niobate HSr_2_Nb_3_O_10_ are sufficiently similar to analogous compounds of the calcium niobate HSr_2_Nb_3_O_10_, as well as Ruddlesden-Popper titanates H_2_Ln_2_Ti_3_O_10_ [16,17,39,40]. Although the synthesis of isostructural hybrids with unbranched amines and alcohols is not particularly difficult and similar compounds are known for many classes of layered oxides, these compounds can serve as precursors for the formation of hybrids with more complex organic molecules. In addition, even such relatively simple systems can exhibit significant physicochemical properties; in particular, it has been shown that hybrid compounds based on normal alcohols and amines exhibit a significantly increased photocatalytic activity in comparison with unmodified analogues in the reaction of hydrogen evolution from aqueous solutions [38,39,40,41,42,43,44]. All this makes the preparation and study of such compounds an urgent task.

## Figures and Tables

**Table 1:** Optimized conditions for the synthesis of inorganic–organic derivatives.

Synthesis of *n*-Alkylamine Derivatives					
Amine	Precursors	Amine Conc., %	Temperature, °C	Duration, d	Solvent for Flushing
MeNH_2_	HSN_3_·yH_2_O	38 (in water)	25	1	Acetone
EtNH_2_		70 (in water)			
PrNH_2_		100			
BuNH_2_		90 (in water)			
HxNH_2_		100	100	3	*n*-Hexane
OcNH_2_		90 (in water)	80	7	
**Synthesis of *n*-Alkoxy Derivatives**					
**Alcohol**	**Precursors**	**Alcohol Conc., %**	**Temperature, °C**	**Duration, d**	**Solvent for Flushing**
MeOH	HSN_3_·yH_2_O	90 (in water)	100	7	Acetone
EtOH		95 (in water)			
PrOH		98 (in water)			
BuOH	HSN_3_ × PrOH		130		
HxOH		100	100		*n*-Hexane
DcOH			180		

**Table 2:** Data on composition and light absorption of the initial protonated niobate and its amine HSr_2_Nb_3_O_10_·xRNH_2_·yH_2_O and alkoxy H_1−x_Sr_2_Nb_3_O_10−x_·xRO·yH_2_O derivatives.

Sample	Total Mass Loss on the TG Curve, %	Composition		Light Absorption	
		x	y	E_g_, eV	λ_max_, nm
HSN_3_·yH_2_O	5.13	−	1.30	3.29	377
×MeNH_2_	6.20	0.95	0.10	3.29	377
×EtNH_2_	9.50	1.00	0.55	3.30	376
×PrNH_2_	11.40	1.00	0.55	3.31	375
×BuNH_2_	13.10	1.00	0.50	3.32	373
×HxNH_2_	16.30	1.00	0.45	−	−
×OcNH_2_	19.90	1.05	0.35	3.32	373
×MeOH	4.00	1.00	0.10	3.22	385
×EtOH	6.30	1.00	0.20	3.25	382
×PrOH	8.30	1.00	0.20	3.27	379
×BuOH	9.70	0.95	0.15	3.30	376
×HxOH	13.30	0.95	0.25	−	−
×DcOH	19.10	0.95	0.05	3.34	371

## Data Availability

The data presented in this study are available in the article.

## Supplementary Materials

The following supporting information can be downloaded at: https://www.mdpi.com/article/10.3390/molecules28124807/s1, Figure S1: XRD patterns of the initial alkaline niobate KSN_3_ and its protonated form HSN_3_·yH_2_O; Figure S2: IR spectra of the initial niobate HSN_3_·yH_2_O and inorganic–organic derivatives with *n*-amines HSN_3_ × RNH_2_ and *n*-alcohols HSN_3_ × ROH; Figure S3: Diffuse reflectance spectra (gray) and corresponding Tauc plots (black) for the initial protonated niobate HSN_3_∙yH_2_O and its derivatives with *n*-amines HSN_3_ × RNH_2_ and *n*-alcohols HSN_3_ × ROH; Figure S4: Thermo-XRD data for the initial protonated niobate HSN∙yHO and its derivatives *n*-amines and *n*-alcohols.

## Sample Availability

Samples of the compounds are available from the authors.
