# Supplementary material for: Synthesis and Characterization of Inorganic-Organic Derivatives of Layered Perovskite-like Niobate HSr2Nb3O10 with n-Amines and n-Alcohols
Source: Molecules. 2023 Jun 16;28(12):4807. doi: 10.3390/molecules28124807 (PMC10304179; doi:10.3390/molecules28124807)
Supplement: Supplementary file 1 [file molecules-28-04807-s001.zip › molecules-2384666-SI.pdf]

Supplementary materials

Article

Synthesis and Characterization of Inorganic-Organic Derivatives of Layered Perovskite-like Niobate $HSr_2Nb_3O_{10}$ with *n*-Amines and *n*-Alcohols

Alina D. Khramova, Oleg I. Silyukov *, Sergei A. Kurnosenko, Ekaterina N. Malygina and Irina A. Zvereva

Department of Chemical Thermodynamics and Kinetics, Institute of Chemistry, Saint Petersburg State University, 198504 Saint Petersburg, Russia; st062003@gmail.com (A.D.K.); st040572@student.spbu.ru (S.A.K.); st805605@student.sbpu.ru (E.N.M.); irina.zvereva@spbu.ru (I.A.Z.)

* Correspondence: oleg.silyukov@spbu.ru

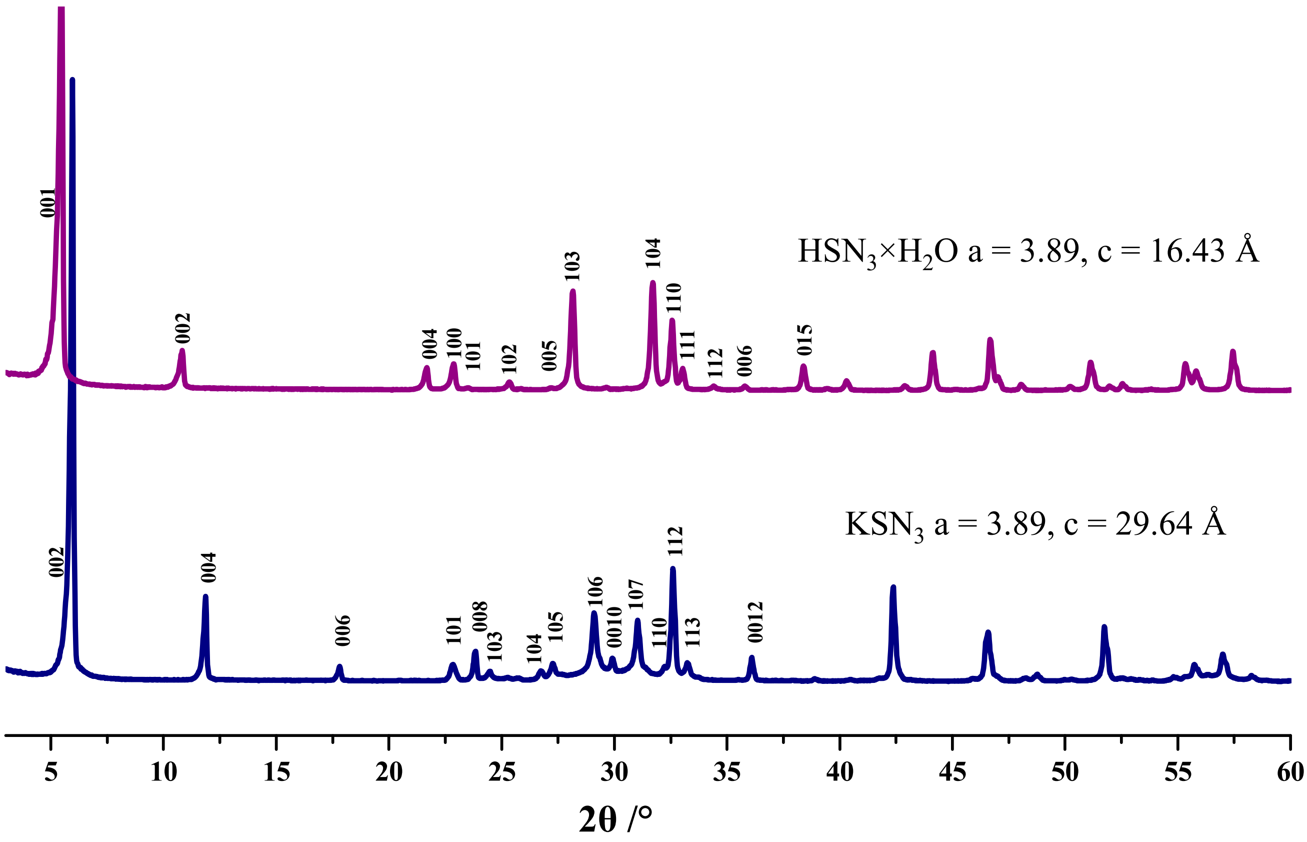

Figure S1. XRD patterns of the initial alkaline niobate KSN_3 and its protonated form $HSN_3{\cdot}yH_2O$

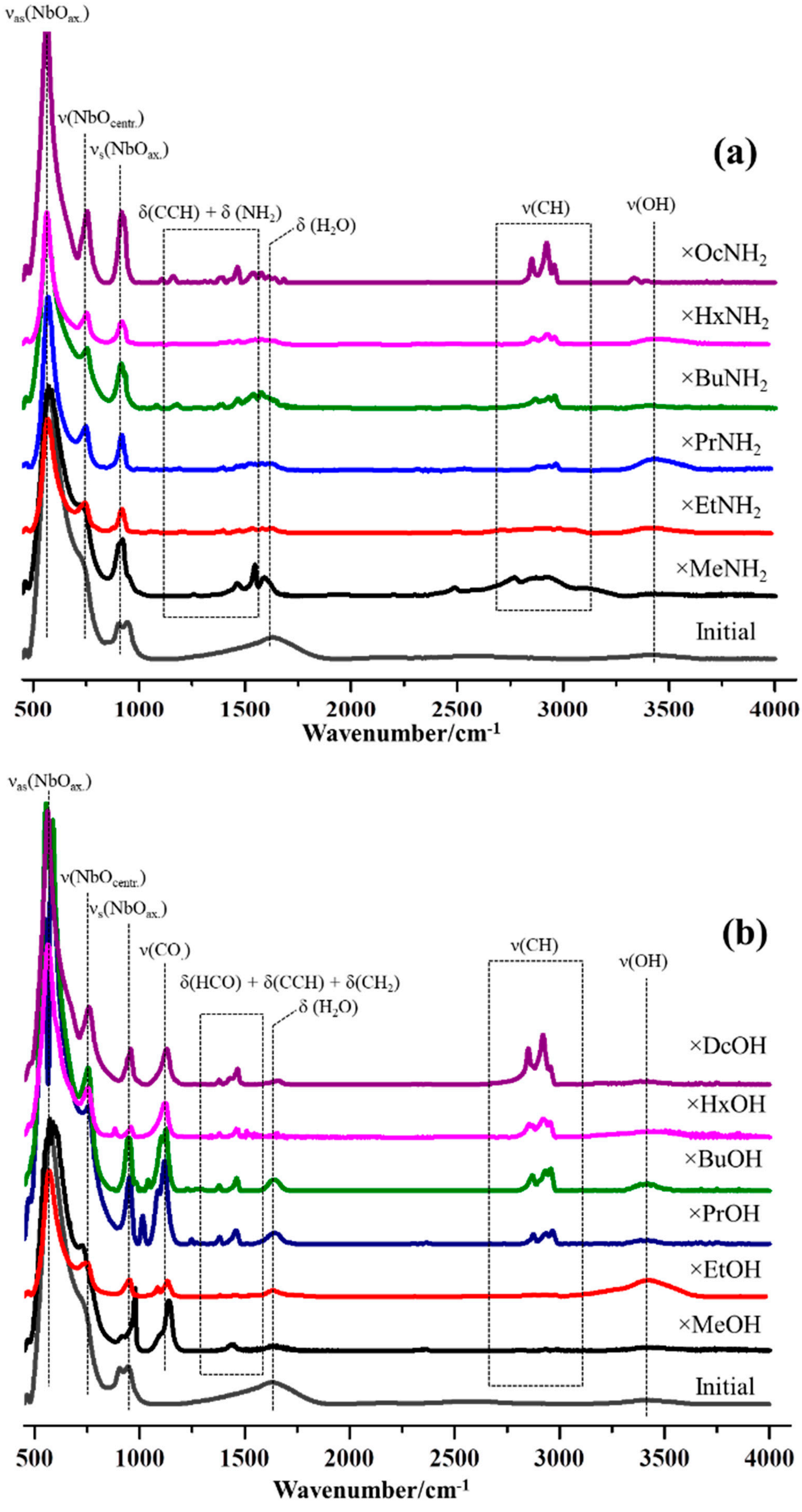

Figure S2. IR spectra of the initial niobate $HSN_3{\cdot}yH_2O$ and inorganic-organic derivatives with *n*-amines $HSN_3{\times}RNH_2$ (a) and *n*-alcohols $HSN_3{\times}ROH$ (b)

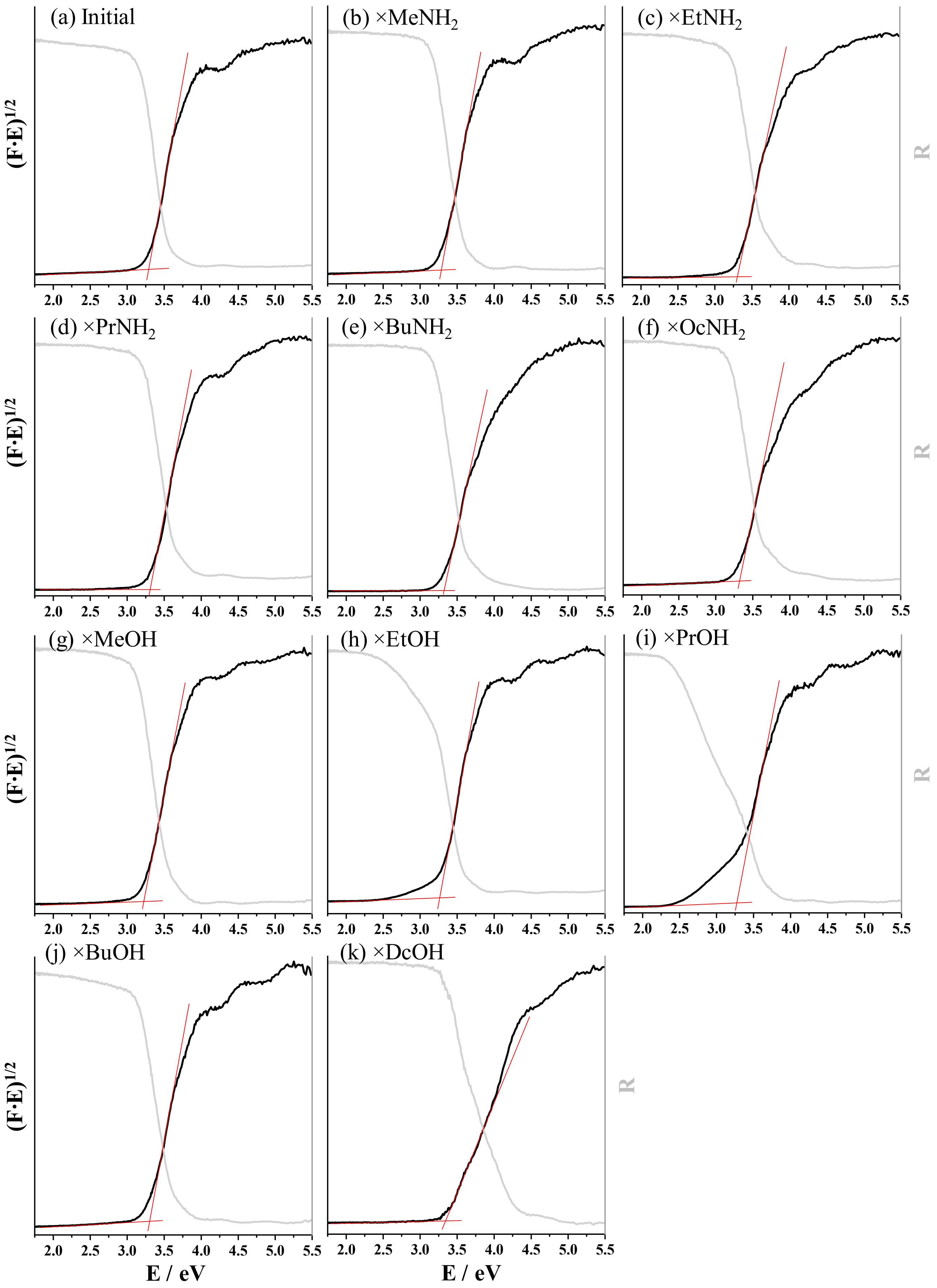

Figure S3. Diffuse reflectance spectra (gray) and corresponding Tauc plots (black) for the initial protonated niobate $HSN_3{\cdot}yH_2O$ (a) and its derivatives with *n*-amines $HSN_3{\times}RNH_2$ (b–f) and *n*-alcohols $HSN_3{\times}ROH$ (g–k)

(a) Initial

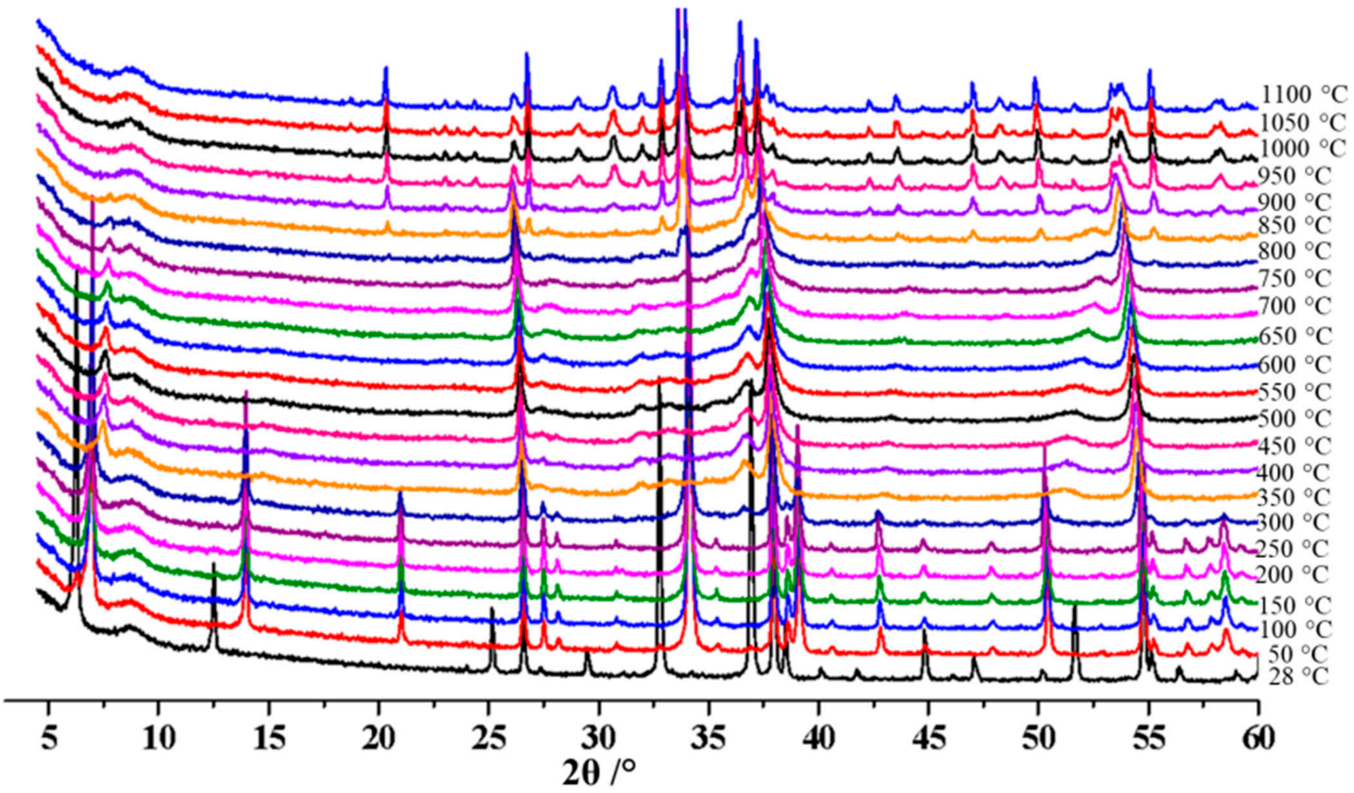

(b) ×$MeNH_2$

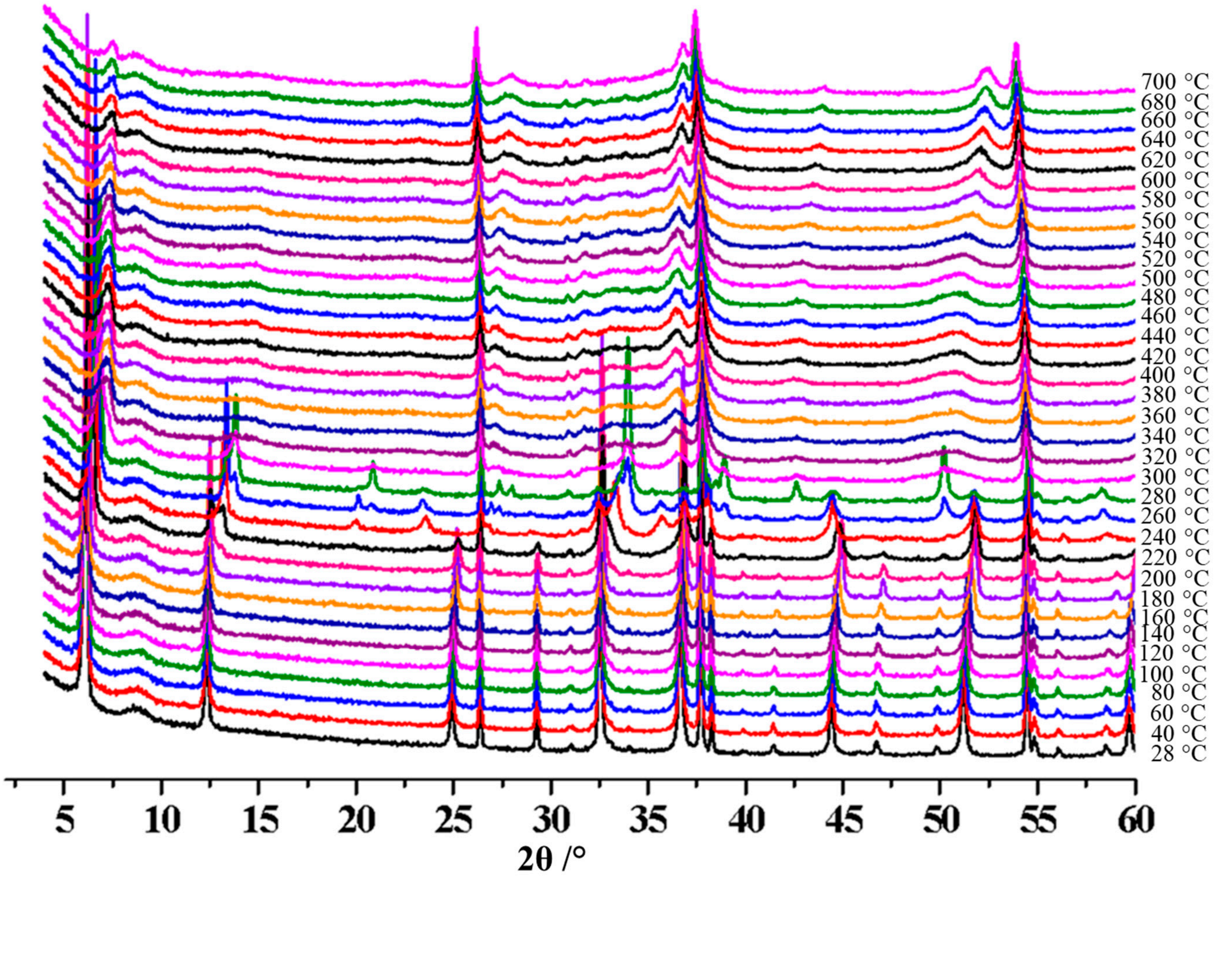

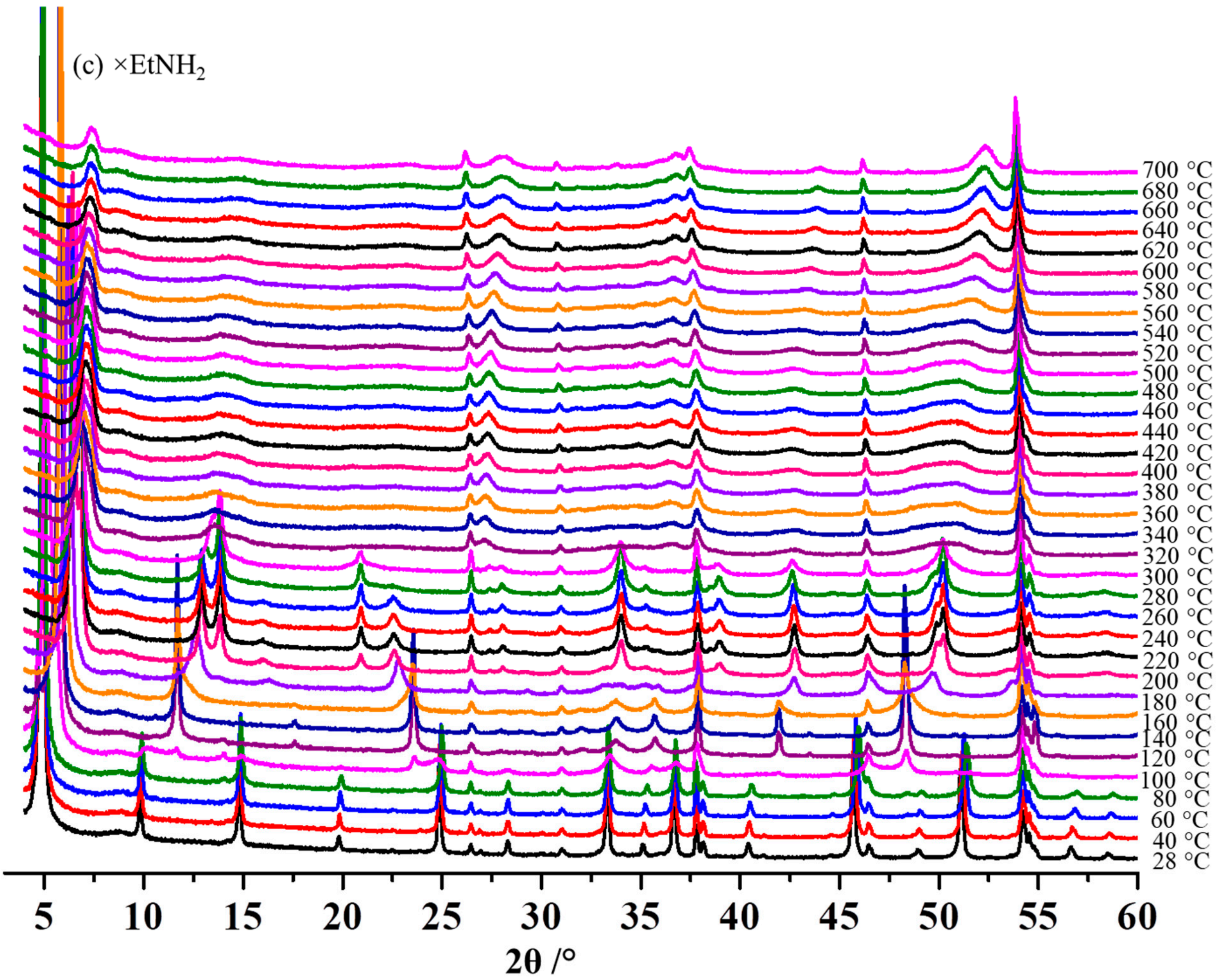
(c) ×EtNH$_2$
700 °C
680 °C
660 °C
640 °C
620 °C
600 °C
580 °C
560 °C
540 °C
520 °C
500 °C
480 °C
460 °C
440 °C
420 °C
400 °C
380 °C
360 °C
340 °C
320 °C
300 °C
280 °C
260 °C
240 °C
220 °C
200 °C
180 °C
160 °C
140 °C
120 °C
100 °C
80 °C
60 °C
40 °C
28 °C
5
10
15
20
25
30
35
40
45
50
55
60
2θ /°

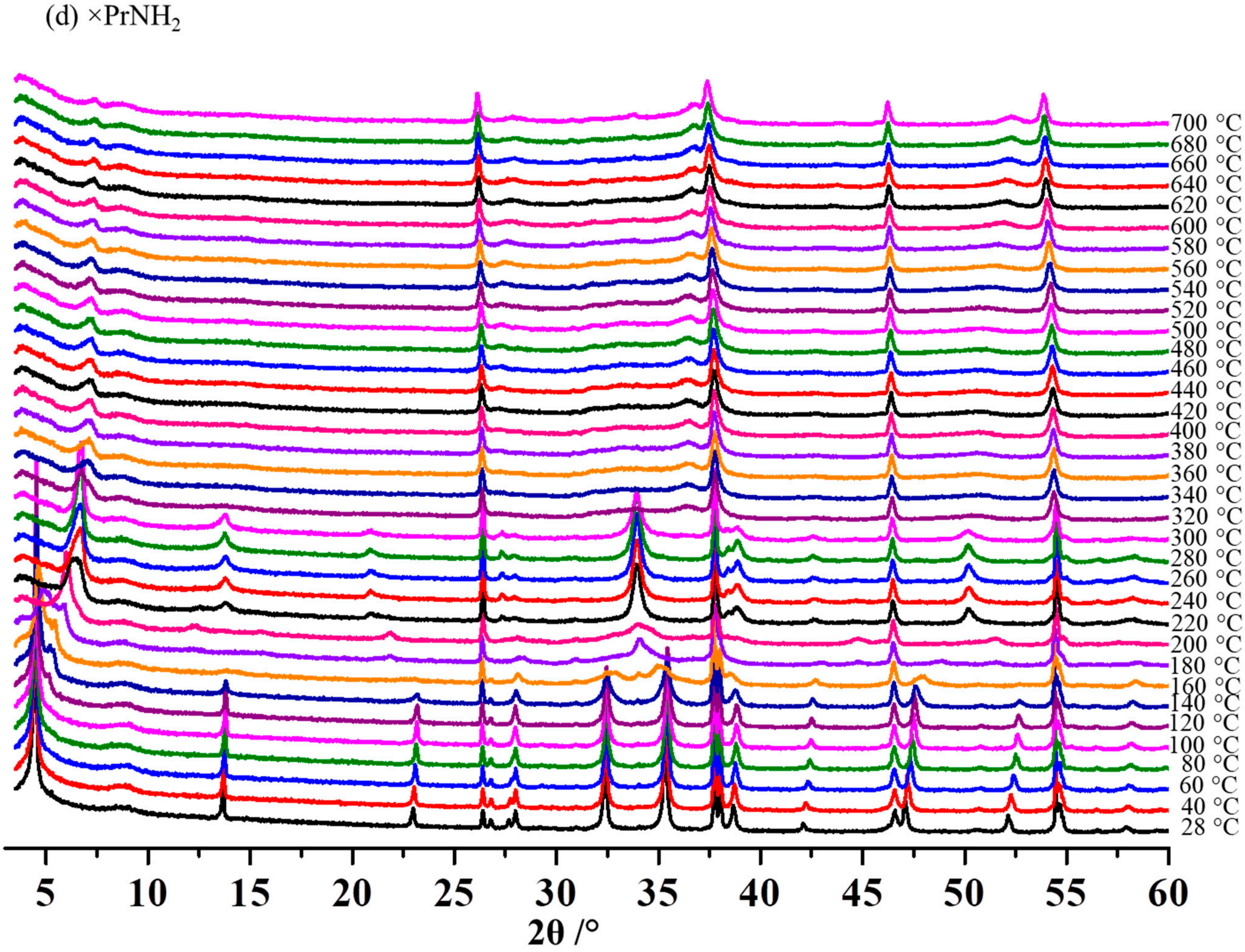
(d) ×PrNH$_2$
700 °C
680 °C
660 °C
640 °C
620 °C
600 °C
580 °C
560 °C
540 °C
520 °C
500 °C
480 °C
460 °C
440 °C
420 °C
400 °C
380 °C
360 °C
340 °C
320 °C
300 °C
280 °C
260 °C
240 °C
220 °C
200 °C
180 °C
160 °C
140 °C
120 °C
100 °C
80 °C
60 °C
40 °C
28 °C
5
10
15
20
25
30
35
40
45
50
55
60
2θ /°

(e) ×BuNH$_2$

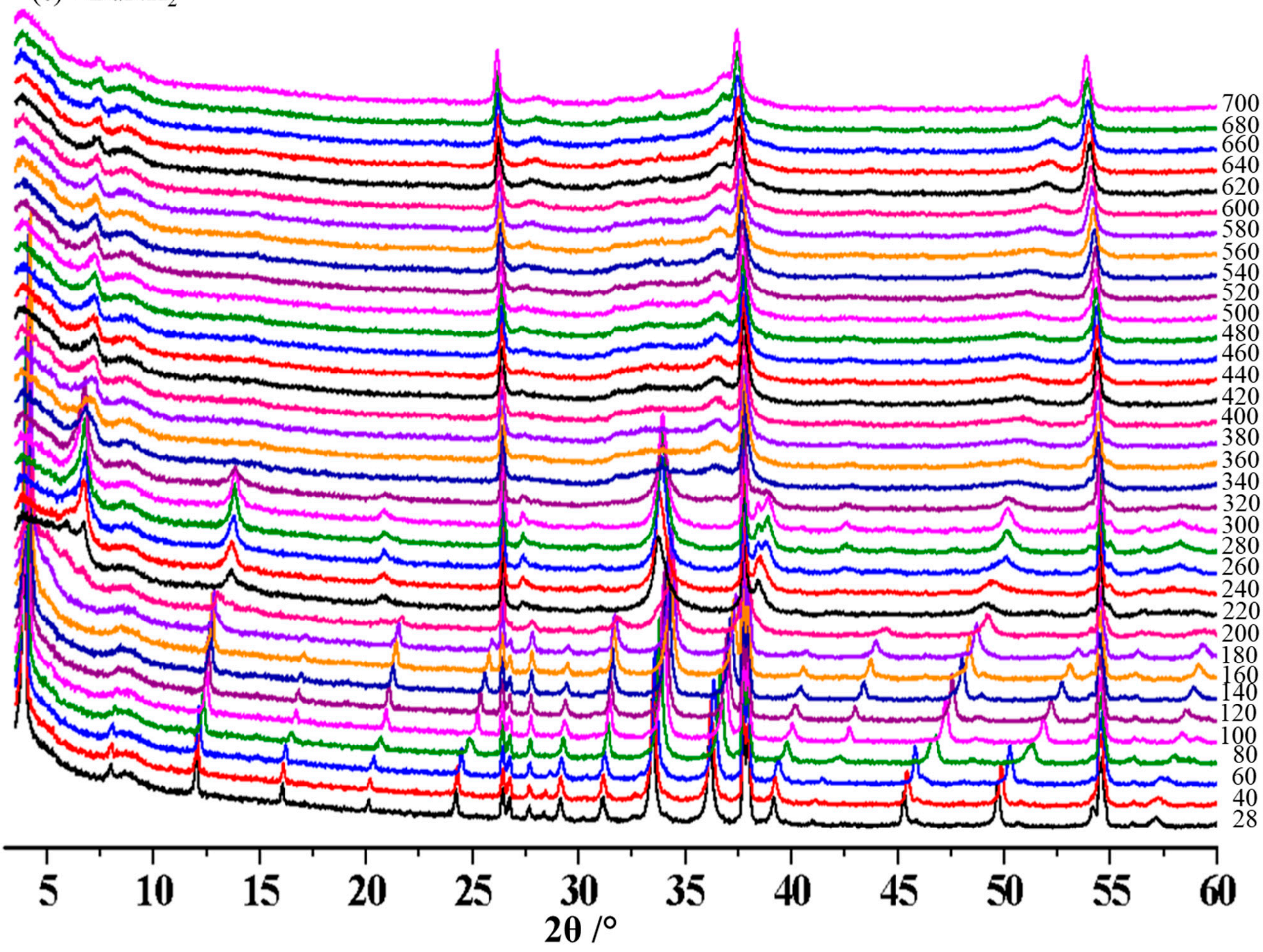

(f) ×MeOH

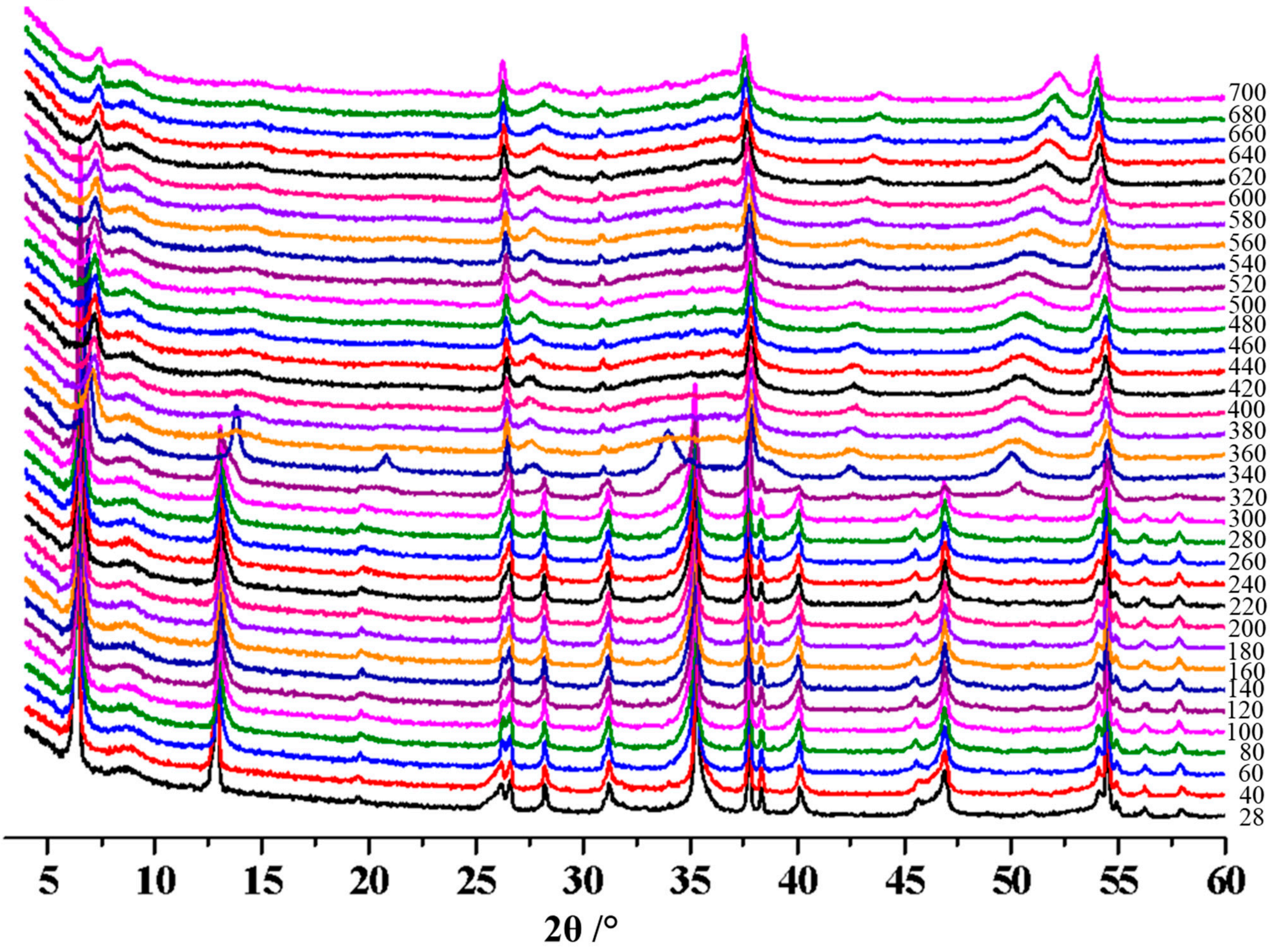

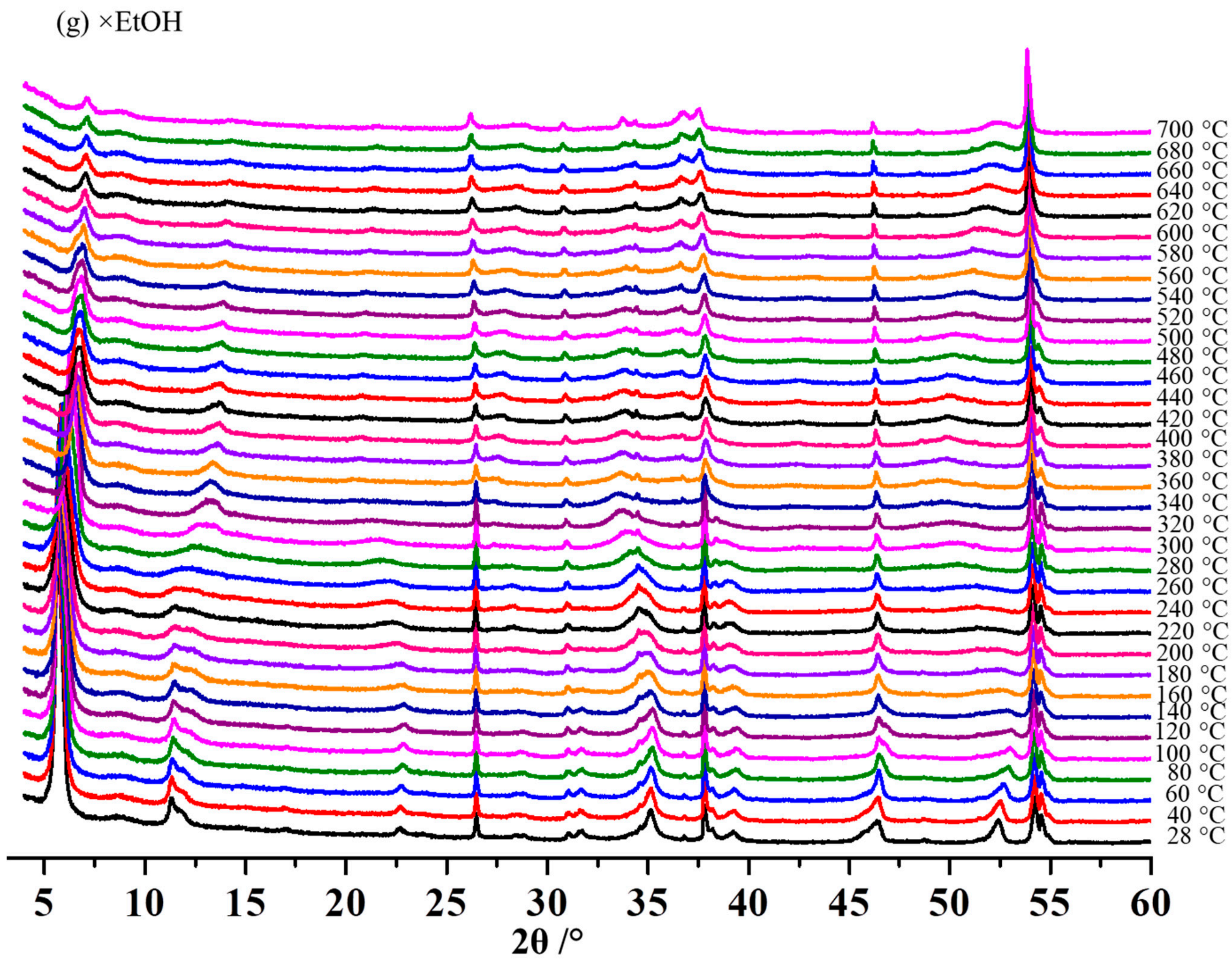
(g) ×EtOH
700 °C
680 °C
660 °C
640 °C
620 °C
600 °C
580 °C
560 °C
540 °C
520 °C
500 °C
480 °C
460 °C
440 °C
420 °C
400 °C
380 °C
360 °C
340 °C
320 °C
300 °C
280 °C
260 °C
240 °C
220 °C
200 °C
180 °C
160 °C
140 °C
120 °C
100 °C
80 °C
60 °C
40 °C
28 °C
5
10
15
20
25
30
35
40
45
50
55
60
2θ /°

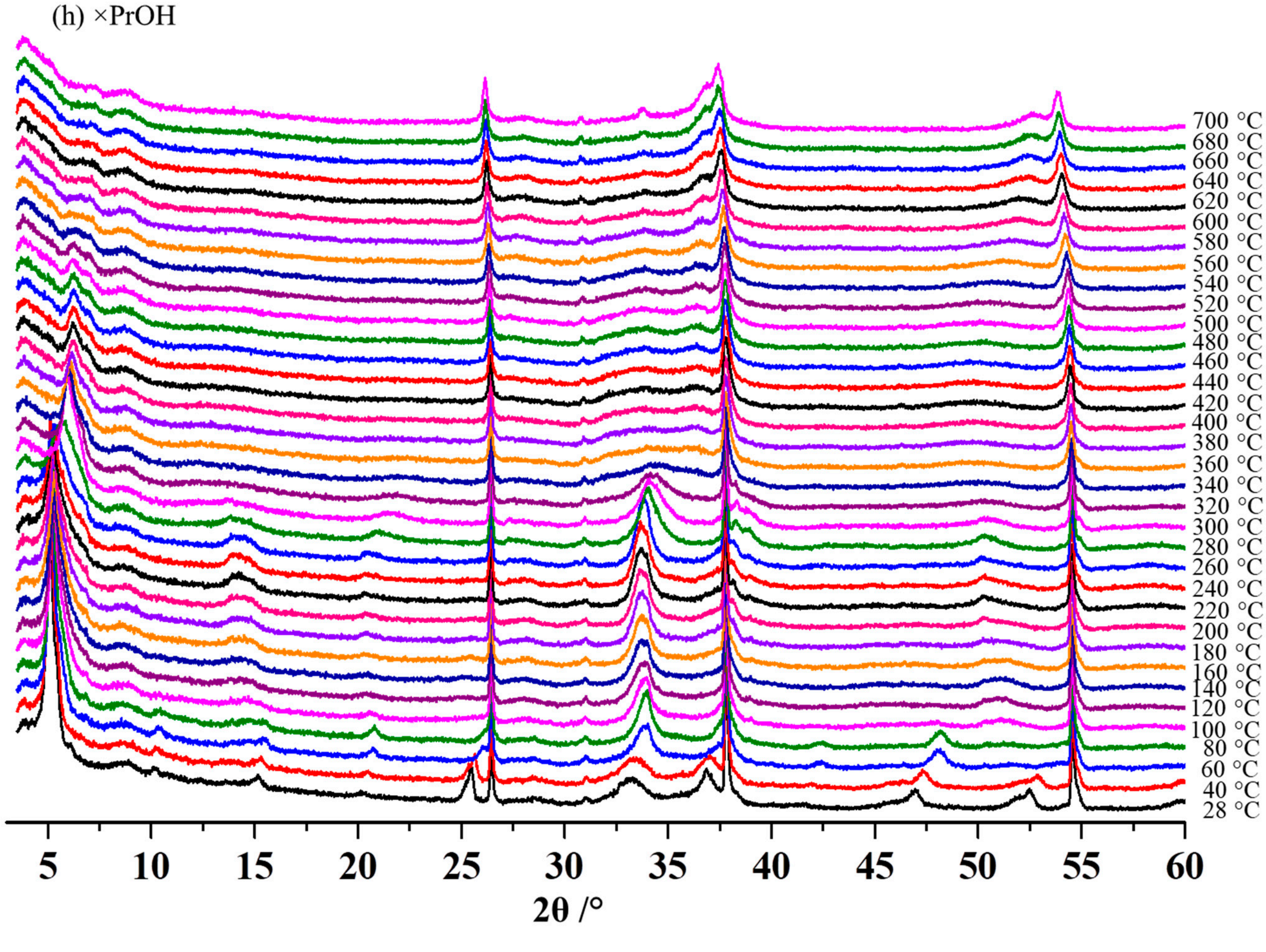
(h) ×PrOH
700 °C
680 °C
660 °C
640 °C
620 °C
600 °C
580 °C
560 °C
540 °C
520 °C
500 °C
480 °C
460 °C
440 °C
420 °C
400 °C
380 °C
360 °C
340 °C
320 °C
300 °C
280 °C
260 °C
240 °C
220 °C
200 °C
180 °C
160 °C
140 °C
120 °C
100 °C
80 °C
60 °C
40 °C
28 °C
5
10
15
20
25
30
35
40
45
50
55
60
2θ /°

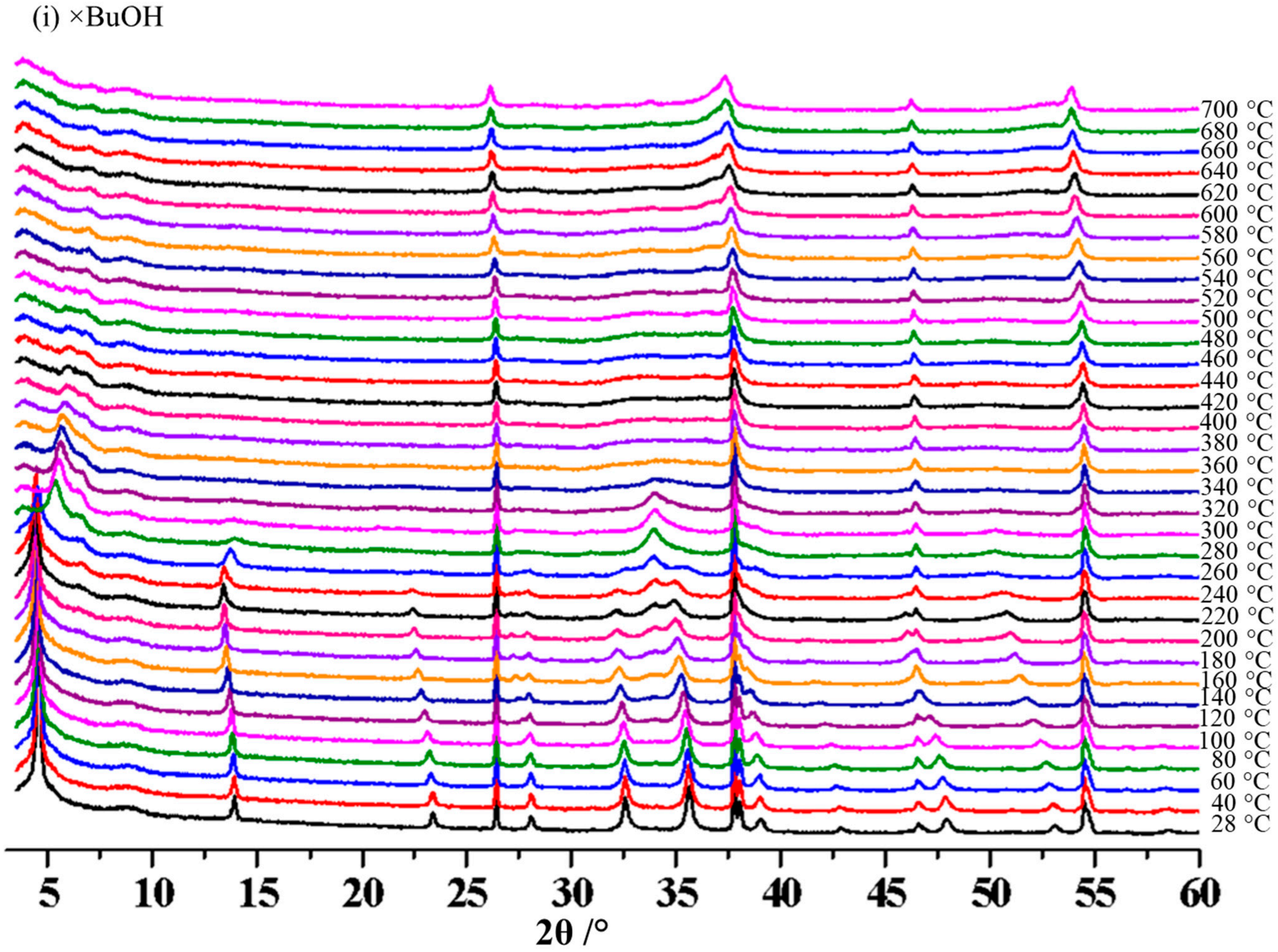

Figure S4. Thermo XRD data for the initial protonated niobate $HSN_3 \cdot yH_2O$ (a) and its derivatives *n*-amines $HSN_3 \times RNH_2$ (b–e) and *n*-alcohols $HSN_3 \times ROH$ (f–i).
